# A Rapid Anterior Auditory Processing Stream through the Insulo-parietal Auditory Field in the Rat

**DOI:** 10.1523/JNEUROSCI.2382-24.2025

**Published:** 2025-08-01

**Authors:** Maciej M. Jankowski, Mousa Karayanni, Mor Harpaz, Ana Polterovich, Israel Nelken

**Affiliations:** ^1^Edmond and Lily Safra Center for Brain Sciences (ELSC), The Hebrew University of Jerusalem, Jerusalem 91904, Israel; ^2^Alexander Silberman Institute of Life Sciences, The Hebrew University of Jerusalem, Jerusalem 91904, Israel; ^3^BioTechMed Center, Multimedia Systems Department, Faculty of Electronics, Telecommunications and Informatics, Gdansk University of Technology, Gdansk 80–233, Poland

**Keywords:** auditory, insular cortex, neuropixels probes, rat, secondary somatosensory cortex, stimulus-specific adaptation

## Abstract

The insular cortex is involved in a wide range of auditory functions in the mammalian brain. We studied the organization and basic response properties of auditory neurons in the insular cortex and adjacent areas by recording responses to sound stimuli in anesthetized female rats. Auditory neurons were present in an insulo-parietal auditory field that spans the boundary between the posterior insula, particularly in the granular insular cortex, and the ventral part of the adjacent nonprimary somatosensory fields. Confirming previous reports, neurons in this field had narrow tuning and were preferentially tuned to relatively low frequencies (<16 kHz). Intriguingly, auditory units in this field displayed shorter minimal latencies than units in the primary auditory cortex, as well as weaker sensitivity to deviance. These results establish the existence of a rapid auditory information stream with distinct properties through the insulo-parietal cortex that may parallel the pathway through the primary and anterior auditory fields.

## Significance Statement

This study unveils a fast auditory processing stream with distinct properties that bypasses the primary auditory cortex in the rat brain. Utilizing neuropixels electrodes, we confirmed the presence of an auditory field at the insulo-parietal junction with an overrepresentation of low frequencies. Neurons in this field have remarkably short response latencies as well as weaker sensitivity to deviant sounds than core auditory areas. This research offers a new perspective on the involvement of insular and parietal cortices in the rapid processing of sounds.

## Introduction

The insular cortex is involved in a wide range of auditory functions in the human brain. Strokes in the human insular cortex are associated with hearing deficits, particularly in temporal resolution (the ability to detect brief gaps between sounds) and sequencing (the ability to correctly order auditory stimuli). Vascular events that include the insulae may result in auditory agnosia to environmental sounds, speech, and music (Spreen et al., 1965; Hyman and Tranel, 1989; Griffiths et al., 1997; Bamiou et al., 2003).

Auditory responses in the human posterior insula resemble those observed in Heschl's gyrus (Zhang et al., 2019). The posterior insula is bilaterally activated when listening to speech, and this activation is believed to be sensory rather than motor (Woolnough et al., 2019). Citherlet et al. (2020) proposed, based on intracranial EEG recordings, that the posterior insula is involved in automatic, preattentive auditory processing that is stimulus-driven and task-independent.

Animal studies also support the sensory role of the posterior insula. In awake squirrel monkeys, auditory neurons were found in higher numbers in the posterior than in the anterior insula, and their response latencies are consistent with direct connections from the medial geniculate body (MGB; Sudakov et al., 1971). Primate MGB projects to the junction between the temporal lobe and the insula throughout its extent (Burton and Jones, 1976). In rhesus monkeys, the posterior insula has been identified as a predominantly auditory region with representations of conspecific communication sounds (Remedios et al., 2009).

These results share two striking properties. Auditory responses in the posterior insula have short latencies and significant similarities with the auditory cortex. Yet, the posterior insula also differs from the auditory cortex, seemingly being involved with vocal communication.

Less is known about auditory processing in the human and primate parietal somatosensory cortex. The stimulation of the human parietal operculoinsular region, where the secondary somatosensory cortex and the posterior insula meet, produces auditory hallucinations (Job et al., 2016; Jaroszynski et al., 2022a). Disruption of auditory–somatosensory integration in this region may result in phantom perceptions, such as tinnitus (Jaroszynski et al., 2022b). In some patients with somatosensory thalamic infarctions, certain sounds produced an intense somatosensory tingling sensation, accompanied by a threefold increase in the responses to sounds in the parietal operculum (secondary somatosensory cortex; Beauchamp and Ro, 2008). Recent data confirmed auditory responses in the human somatosensory cortex (Pérez-Bellido et al., 2018; Rahman et al., 2020). In primates as well, some neurons recorded in the secondary somatosensory cortex responded to sounds (Lemus et al., 2010; Hihara et al., 2015).

In rats and mice, the posterior insula is innervated by the MGB and contains auditory neurons (Rodgers et al., 2008; Kimura et al., 2010; Sawatari et al., 2011; Guo et al., 2012; Gogolla et al., 2014; Takemoto et al., 2014; Tsukano et al., 2017). In rats, short-latency auditory responses were detected in both primary and nonprimary somatosensory fields (Brett-Green et al., 2003, 2004; Wallace et al., 2004; Maruyama and Komai, 2018).

Importantly, in rats, the auditory-responsive area in the posterior insula is anatomically separated from other auditory fields, unlike in mice (Rodgers et al., 2008; Kimura et al., 2010; Guo et al., 2012). The auditory responses in rat insular and somatosensory cortices, together with their anatomical separation from the rest of the rat auditory cortex, make the rat into an excellent model for studying these fields.

Here, we studied the organization of the auditory activity in the posterior insula and the adjacent nonprimary somatosensory cortex in anesthetized rats. We recorded under anesthesia in order to study the sensory aspects of the auditory responses (Wong et al., 2004; Remedios et al., 2009; Citherlet et al., 2020). We delineate an auditory field that spans the border between the posterior insula and the adjacent parietal nonprimary somatosensory cortex. Neurons in this field form a sensory representation that is distinct from that of the core auditory areas, with surprisingly short latencies and weaker sensitivity to auditory context.

## Materials and Methods

### Animals

Thirty female Sabra rats (240–325 g) that were naive to the experiments were used from Envigo. The animals were housed in pairs per cage and had unrestricted access to water and standard rodent food prior to the experiments. The rats were maintained in a temperature and humidity-controlled room on a 12 h light/dark cycle with lights on from 7:00 A.M. to 7:00 P.M. The experiments were carried out in compliance with the regulations of the ethics committee at The Hebrew University of Jerusalem, an institution that is accredited by the Association for Assessment and Accreditation of Laboratory Animal Care (ethics approval number NS-18-15652-4).

### Electrodes and target brain structures

Extracellular recordings were conducted in the posterior insular cortex (Ins) and adjoining nonprimary somatosensory fields [S2 in Paxinos and Watson (2007), including multiple functional fields (Fabri and Burton, 1991, Remple et al., 2003)]. We used two types of electrodes: tungsten electrode arrays (in seven rats) and Neuropixels silicon probes (in eight rats; Table 1). These data were compared with neuronal activity recorded in three structures of the ascending auditory system using Neuropixels electrodes in additional five rats per structure: primary and secondary auditory cortices (A1 and AC), MGB, and inferior colliculus (IC). The responses to other stimuli collected in these latter experiments have been previously published (Harpaz et al., 2021; tone clouds).

**Table 1.** A breakdown of the number of electrode penetrations and number of recorded units per animal

Animal number/experiment date	Total number of penetrations	Total number of units	Number of Ins/Par units	Number of AI units	Number of DI units	Number of GI units	Number of Par units
1 (050318)	4	794	452	4	215	233	294
2 (070318)	3	793	345	0	114	231	337
3 (190318)	3	638	342	19	124	199	269
4 (210318)	3	521	233	7	97	129	199
5 (260218)	3	378	80	0	0	80	298
6 (140318)	1	134	76	0	21	55	58
7 (280218)	2	309	206	14	87	105	103
8 (260318)	3	734	150	0	12	138	353

#### Tungsten electrodes

Two types of custom-built arrays were constructed using monopolar tungsten electrodes (0.6 MOhm, glass-coated, Alpha Omega Engineering). These arrays were built with three or nine tungsten electrodes and were used in two and five rats, respectively. The spacing between electrodes in the three-electrode arrays was 300–500 µm, and in the 3 × 3 electrode arrays, it was 400–600 µm. The arrays were mounted on custom-made microdrives, which allowed for the gentle removal of the electrodes from the fixed brain tissue after transcardial perfusion in order to preserve the shape of the electrode tracks in the brain tissue for precise histological reconstructions. Before each experiment, the electrodes were covered with multiple layers of orange–red fluorescent dye (DiI, Thermo Fisher Scientific, Molecular Probes) dissolved in absolute ethanol (J.T.Baker) and dried. Before and after the experiments, the electrodes were cleaned by washing them with trypsin solution 10× from porcine pancreas (Sigma-Aldrich) and purified water.

#### Neuropixels electrodes

Phase 3A Neuropixels silicon probes (Imec) were used. A thin and flexible ground cable was soldered to the electrode's PCB. The ground and reference contacts were shorted during the experiments. The ground cable was connected to a low-impedance reference contact on the head of the rat through a single gold-plated connector (Mill-Max). The electrodes were washed with trypsin solution 10× and rinsed with purified water before and after each experiment and then placed for ∼1 h in a 4% solution of tergazyme in purified water (Alconox). Finally, the electrodes were carefully washed in purified water. The underside of the silicon substrate of the electrode was coated with DiI dissolved in a 70% isopropanol solution in water (Sigma-Aldrich) and dried before use.

### Surgery

Rats were anesthetized with an initial intramuscular injection of ketamine hydrochloride (∼40 mg/kg, Clorketam 1 g/10 ml, Vetoquinol) and medetomidine hydrochloride (∼0.1 mg/kg, Cepetor 1 mg/ml). The surgical level of anesthesia was verified by the absence of pedal withdrawal and corneal reflexes and by a slow, steady breathing rate. The animal's head and neck were shaved, and body temperature was regulated using a closed-loop heating system controlled by a rectal probe (FHC). The animal was placed on its back, and an incision was made on the neck above the trachea. The trachea was exposed by retracting the fat tissue and muscle using blunt curved tweezers, and a transverse cut was made in the trachea. A stainless steel tube with blunted edges, covered at its end with a 4-mm-long piece of polyethylene tubing (16G, made of injection needle 1.6 × 40 mm; Becton, Dickinson and Company) was inserted into the trachea. The metal tube was tied with a silk suture (Braided Silk Suture 6-S, Teleflex Medical) to the trachea above the thicker polyethylene part. Next, the animal was positioned on its ventral side, and the head was mounted in a custom head holder designed for auditory experiments, holding the animal by the maxilla. The holder was attached to the stereotaxic apparatus (David Kopf Instruments). The free end of the metal tube inserted into the trachea was attached to the tubing of the anesthetic machine with a time-cycled volume ventilator (Hallowell EMC). The animal was ventilated with 100% oxygen. The breathing parameters and CO_2_ level in exhaled gases were monitored using a capnograph (MicroCap, Oridion Medical 1987). One hour after the injection of medetomidine and ketamine, 1% of halothane (Sigma-Aldrich) was added to the inhaled oxygen using a calibrated vaporizer (Matrx VIP 3000, Midmark Corporation). After another half an hour, the halothane level was increased to 1.5% and adjusted individually if needed. The level of anesthesia was kept at a stable level throughout the entire experiment, and the CO_2_ level was maintained between 35 and 45 mmHg. The level of anesthesia used allowed for ventilation without muscle relaxation.

A longitudinal incision of the skin on the head (2–3 cm) was made, and the dorsal and left lateral surfaces of the skull were exposed. The connective tissue covering the bones was removed, and the bones were cleaned with sterile saline. Once the surface of the skull was clean and dry, a target reference point for the chosen recording site was marked on the bone (insulo-parietal field, AP, −1.0 mm; ML, −6.1 mm from the bregma; A1/AC, AP, −5.1 mm; ML, guided by landmarks on temporal and parietal bones; MGB, AP, −5.6 mm; ML, −3.5 mm; IC, AP, −7.1 mm; ML, −2.0 mm). The nonprimary somatosensory fields targeted by this penetration are marked as S2 in the Paxinos atlas (Paxinos and Watson, 2007); this area is likely to contain at least two somatosensory fields, S2 proper and the parietal ventral (PV) area. Since the experiment was purely auditory, we did not try to find the boundary between them. The electrode was inserted into the brain under an angle of 20° to the vertical axis; one penetration was perpendicular to the horizontal plane with the following coordinates: AP, −8.0 mm; ML, −2.0 mm. The soft tissue and bones, except around the implantation site, were covered with acrylic glue and acrylic dental cement (R21, AXIA; Coral-fix). A well for saline was shaped around the implantation site using acrylic dental cement. A bare silver wire soldered to a thin, flexible ground cable was attached firmly with acrylic glue near the posterior edge of the implantation site (bare silver wire 0.01 in, A-M Systems). The craniotomy was created by drilling (square ∼3 × 3 mm).

### Tungsten electrode array insertion

The dura was gently resected. The electrodes were slowly (<10 µm/s) inserted vertically into the brain tissue until auditory units were detected [broadband noise (BBN) stimulus] using a single-axis motorized micromanipulator (S-IVM-1000, Scientifica). After recording neuronal responses to all chosen sound stimulation protocols, the electrodes were slowly advanced deeper into the brain tissue to reach new recording positions. A single brain penetration was performed in each experiment. On average, auditory-responsive units in the insulo-parietal field were most frequent between 3.4 and 3.8 mm below the brain surface. At the end of the experiment, the end positions of electrodes in the brain were preserved during tissue fixation, making it possible to perform a precise histological reconstruction of the electrode tracks. For that purpose, the small microdrive with the electrode array was fixed to the skull with acrylic dental cement (Coral-fix). Once the cement cured, the microdrive was released from the micromanipulator. The rat was transferred to a fume hood for transcardial perfusion with the electrodes remaining in the brain.

### Neuropixels probe insertion

The 0.3–0.5-mm-long slot in the dura was gently resected. The Neuropixels probe (phase 3A) was slowly (<10 µm/s) inserted into the brain tissue while avoiding visible blood vessels to minimize trauma. A single-axis motorized micromanipulator (S-IVM-1000, Scientifica) was used for insertion. Multiple electrode insertions (2–6) were made around the target coordinates. Neuronal responses to sounds were recorded with “bank 0” of the electrode—the 384 most distal electrodes (arranged over ∼3.8 mm of the shank). The electrode targeting the insulo-parietal field was inserted ∼4.2–5.1 mm below the brain surface. BBN and high-level tones (70–80 dB SPL) were used to determine the presence of auditory responses. Whenever auditory-responsive units were found, the full set of sound stimulation protocols was presented. Protocols were recorded in 23 out of 30 penetrations in Ins; 10 of 16 penetrations in A1/AC; 8 of 9 penetrations in MGB; and 8 of 9 penetrations in IC. At the end of the experiment, the Neuropixels probe was removed from the brain tissue, and the rat was transferred to a fume hood for transcardial perfusion.

### In vivo electrophysiology

Tungsten electrode recordings were performed using the AlphaLab SnR recording system (Alpha Omega Engineering). The raw voltage traces, captured at a sampling rate of 22 kHz, were then processed off-line using MATLAB (The MathWorks). Neuropixels silicon probes were connected to a dedicated recording system (Phase 3A) as outlined on the website: https://github.com/cortex-lab/neuropixels/wiki/About_Neuropixels. Filtered and preprocessed voltage traces for action potentials (sampled at 30 kHz, filtered with a high-pass filter set at 300 Hz) and local field potentials (sampled at 2.5 kHz) were recorded and subsequently underwent further off-line analysis using MATLAB.

### Auditory stimulation

The recordings were conducted in a soundproof chamber (IAC). Audio stimuli were generated using a custom-written MATLAB (The MathWorks). Sound signals were converted to voltage signals at a sampling rate of 192 kHz by a sound card (RME HDSP 9632), attenuated [using a PA5 programmable attenuator, Tucker Davis Technologies (TDT)], and played through a two-channel electrostatic speaker driver (ED1, TDT) and an electrostatic speaker with coupler (EC1, from TDT). The speaker delivered sound through a flexible PVC tube placed in the right ear canal. The system was calibrated using a Knowles miniature microphone (EK3133-000, manufactured by Knowles, and calibrated relative to a B&K 0.25″ microphone) in the ears of several test rats. For pure tones, the attenuation level of 0 dB corresponded to 100 ± 10 dB SPL in the frequency range 1–30 kHz.

### Sounds and stimulation protocols

#### BBN

Responses to BBN (bandwidth, 0–50 kHz) were collected by using a sequence of 280 bursts, each with a duration of 200 ms, 10 ms linear onset and offset ramps, an interstimulus interval (ISI; onset-to-onset) of 500 ms, and seven different attenuation levels (0–60 dB at 10 dB steps). Bursts at different attenuation levels were presented in a pseudorandom manner, so that each sound level was presented 40 times.

#### Frequency response

Frequency response areas (FRAs) were determined by presenting quasirandom frequency sequences of 370 pure-tone bursts (with a duration of 50 ms, a 5 ms rise/fall time, and an ISI of 500 ms) at 37 frequencies (ranging from 1 to 64 kHz, with frequencies equally spaced on a logarithmic frequency scale, 6/octave, 10 repeats each). The 370 pure-tone bursts were first presented at 0 dB attenuation, then again at levels that decreased in 10 dB steps until the threshold of neural activity was reached (usually at 50–70 dB attenuation). These data were used to determine the basic properties of auditory responses and to select a test frequency (TF)—a frequency that gave rise to the most consistent multiunit responses at all sound levels. If activity was recorded from several neurons simultaneously, the minimum response threshold and TF were selected to match either the neuron with the most pronounced responses to pure tones or the most common TF and minimum response threshold among all recorded units. During recordings with Neuropixels silicon probes, one to three TFs were chosen per penetration based on the most prominent responses, and the rest of the protocol was presented multiple times, once for each TF.

#### Pure-tone sequences

All sequences consisted of 500 tone presentations, with an ISI of 300 ms. Each tone had a duration of 30 ms with 5 ms linear onset and offset ramps. The tones were presented at a constant level (usually ∼20 dB above the minimum threshold at the TF). Frequencies *f*1 and *f*2 (at a frequency separation Δ*f* = *f*2/*f*1 = 1.44) were centered at the TF. However, since the different units recorded simultaneously could have different frequency tuning, for many units, *f*1 and *f*2 were not centered on their best frequency.

Seven test sequences that included frequencies *f*1 and *f*2 were used to study the habituation profiles of insular cortex units.

1. In one oddball sequence, *f*1 occurred 475 times (95%, standard) and *f*2 25 times (5%, deviant); in the other, the roles of *f*1 and *f*2 were reversed.
2. In the “rare” sequences, 475 trials (95%) consisted of silence only, and 25 trials (5%) had tones of frequency *f*1 or *f*2, respectively.
3. The “equal” sequence contained equal numbers of *f*1 and *f*2 (50%, 250 presentations each).
4. Two multitone sequences were recorded, called “diverse broad” (12 equally spaced frequencies that included *f*1 and *f*2) and “diverse narrow” (20 equally spaced frequencies covering a total range of ∼1 octave).

The diverse broad sequence contained tones of frequencies *f*1 and *f*2 with a probability of 5% (25 times each). The other 450 stimuli of the sequence consisted of 10 different frequencies (presented 45 times each, with a 9% probability). These additional 10 tone frequencies were distributed below *f*1 and above *f*2, with successive frequencies separated by the same interval as *f*1 and *f*2 (frequency ratio of 1.44). The use of only 12 frequencies was necessary to avoid exceeding the frequency range of 1–64 kHz. To the extent possible, frequencies *f*1 and *f*2 were positioned in the middle of the frequency range used. Their position in the sequence was shifted when necessary to ensure that the overall range of frequencies tested did not exceed 1–64 kHz. In the diverse narrow sequence, tones of frequencies *f*1 and *f*2 were played together with 18 other tones, 25 times each. The 20 tones had logarithmically spaced frequencies with a ratio between the lowest and the highest tone in the sequence being 2.16 (slightly more than twice the distance between *f*1 and *f*2). These values were selected so that *f*1 was the 6th and *f*2 the 15th frequency in this sequence.

### Histological analyses

Unconscious animals received a lethal injection of sodium pentobarbital (900 mg i.p. per rat, pentobarbital sodium 200 mg/ml, CTS Chemical Industries). When the rats stopped breathing, they were transcardially perfused in a fume hood with 350 ml of 0.1 M phosphate-buffered saline (PBS, Sigma-Aldrich) at room temperature, followed by 400 ml of 7% formaldehyde in 0.1 M PBS at ∼4°C (formaldehyde 35% w/w, Bio-Lab). After perfusion, the brains were removed from the skulls and placed in 7% formaldehyde in 0.1 M PBS for at least 72 h at ∼4°C. In rats implanted with tungsten electrode arrays, before removing the brain from the skull, the electrodes were slowly withdrawn from the fixed brain tissue using the small microdrive still attached to the skull. Following fixation in formaldehyde, the brains were transferred to 30% sucrose, 4% formaldehyde solution in 0.1 M PBS at ∼4°C (Sigma-Aldrich) for cryoprotection for ∼7–14 d. The brains were leveled, and a small triangular longitudinal cut on the right hemisphere cortex was made (hemisphere marker). Each brain was placed in a polyethylene embedding mold (Peel-A-Way, Sigma-Aldrich). The mold was filled with optimal cutting temperature compound (OCT compound, Tissue-Tek). The polyethylene mold with the brain and OCT was placed in a custom aluminum freezing mold filled with a small amount of absolute ethanol (J.T.Baker). The aluminum was then filled with dry ice to ensure that the brain froze rapidly. Frozen blocks with the leveled brains were transferred to a cryostat (CM1950, Leica). Consecutive 35-µm-thick coronal sections were cut around the implantation site (left hemisphere). Brain slices were stained with green fluorescent Nissl stain (NeuroTrace 500/525 Green Fluorescent Nissl Stain, Molecular Probes) according to the manufacturer's instructions. The slices were then mounted onto slides (three slices per slide) and, after drying, covered with a mounting medium (Vectashield H1200, Vector Laboratories) and a cover glass. The slides were examined under standard fluorescent and bright-field microscopy with 4× and 10× objectives. Multi-image array pictures were taken and arranged in a series of all consecutive sections around the implantation sites, from the most anterior to the most posterior. The positions of the electrode tips were estimated on the most relevant histological sections, and recording positions were proportionally derived.

### Quantification and statistical analysis

#### Preprocessing

Twenty-two sessions of Neuropixels recordings were analyzed for this study. Each session was initially sorted using Kilosort (Pachitariu et al., 2016) and then manually curated using phy (https://github.com/kwikteam/phy). In 11 of 23 penetrations, the noncurated units were first analyzed to look for auditory-responsive units. These were used to determine the extent along the shank in which auditory activity was present, and only units in that region were manually curated.

#### Localization of Neuropixels-recorded units

**Figure 1.**
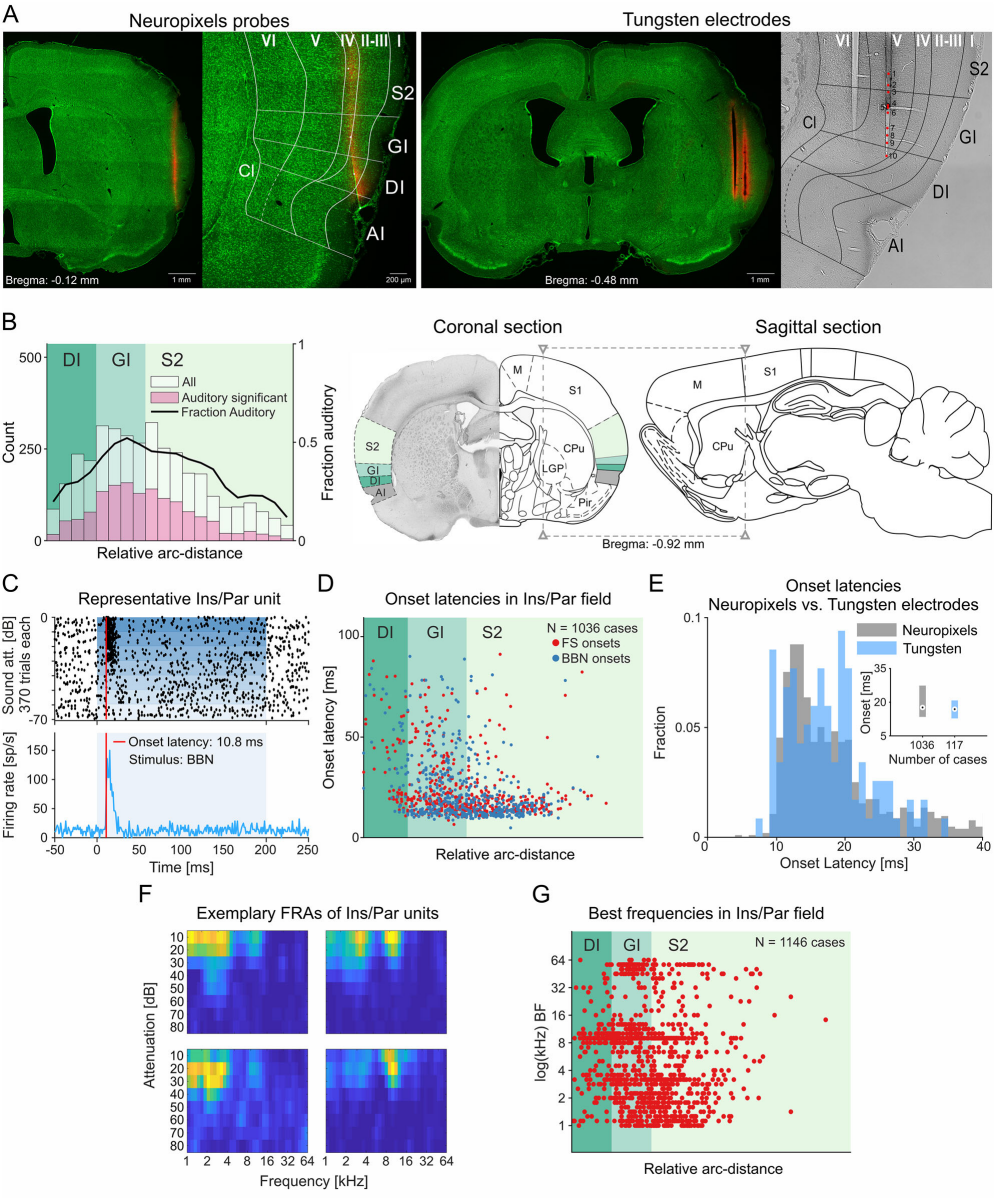
Location, onset latencies, and frequency tuning of auditory neurons in the insulo-parietal cortical field. ***A***, Representative coronal brain sections with electrode tracks through Ins and the adjacent somatosensory fields for Neuropixels (left) and tungsten electrodes (right). An enlarged view of the region of interest (ROI) is shown with Ins subregions (GI, DI, AI) and cortical layers. The recording locations for tungsten electrodes were marked with red dots on the bright-field image of the ROI. Brain sections were stained with fluorescent green Nissl's stain, and electrodes were coated with DiI for better identification of the track. In this figure, S2 serves as a shorthand for the nonprimary somatosensory fields adjacent to the insula. ***B***, Probability of occurrence of auditory neurons recorded with Neuropixels probes in insulo-parietal field. The majority of auditory units were found in the GI and the ventral part of the somatosensory fields, most likely corresponding to somatosensory field PV. The *x* axis displays anatomical location in terms of the arc distance normalized to the atlas subdivisions on the right sections. The right *y* axis displays fraction auditory values, which are calculated by the proportion of auditory units relative to the total units recorded within each arc-distance bin and are smoothed with a square filter the length of two arc-distance bins. The atlas sections also show the approximate lateral and anteroposterior location of the insulo-parietal auditory field in rat brain (see Materials and Methods for details of the computation of the arc-distance values). ***C***, A raster plot and histogram showing the responses of a representative unit from the insulo-parietal auditory field (Ins/Par) to BBN, characterized by a short-onset latency (10.8 ms). Sound levels increase from top to bottom. ***D***, Onset latencies for all responding units recorded with Neuropixels probes in response to frequency sweeps (FS) and BBN. Large numbers of GI and S2 units had short-onset latencies. ***E***, Comparison between onset latencies of units recorded with Neuropixels versus tungsten electrodes showed comparable results for both techniques. ***F***, FRAs of exemplary units recorded in insulo-parietal field showing BF < 16 kHz. ***G***, Best frequencies (BFs) for all units recorded with Neuropixels probes. The BFs < 16 kHz were dominating. I, cortical layer 1; II–III, cortical layers 2/3; IV, cortical layer 4; V, cortical layer 5; VI, cortical layer 6; AI, agranular insular cortex; Cl, claustrum; CPu, caudate–putamen; DI, dysgranular insular cortex; GI, granular insular cortex; LGP, lateral globus pallidus; M, motor cortex; Pir, piriform cortex; S1, primary somatosensory cortex; S2, nonprimary somatosensory fields.

To locate the Neuropixels-recorded units within the brain, a third-order polynomial was fitted to the shank trajectory on images of the histological sections, and units were located on the histological sections based on their relative location along the shank. In order to standardize the locations of the Neuropixels-recorded units along the insular-parietal field, we performed a linear transformation of the channel coordinates on the shank. This transformation set the point on the shank corresponding to the border between DI and GI as 0 and the border between GI and the somatosensory fields as 1. These linearly normalized distance values along the shank are termed “arc distance” in the manuscript (Fig. 1*B*) and allow us to compare unit locations consistently across different recordings.

#### Selection of responsive neurons

For inclusion in the localization, BFs, and onsets analyses, units were required to emit a minimal number of spikes (30 spikes in total for the BBN and FS protocols; spikes were counted over an interval from 100 ms before until 100 ms after the stimulus).

Response significance was determined by a Poisson likelihood ratio test between a homogenous Poisson process whose rate was equal to the average activity during the 100 ms preceding stimulus onset and an inhomogeneous Poisson process with rates that were computed in 10 windows of 10 ms each, for a total of 100 ms following stimulus onset. The logarithms of the probability ratios were summed to form a log likelihood (LL) ratio, under the null hypothesis, 2LL ∼ *χ*^2^ (10).

A unit was considered as responsive when it was active (as defined above) and had significant responses (*p* < 0.01). For the insulo-parietal NPX dataset, we also included units whose responses were significant to one of the frequencies used in the rare condition with the more stringent significance threshold of 0.005 (Bonferroni’s correction for a *p* value of 0.01 with two tests).

#### Onset latencies

Trial-averaged responses were smoothed using a square filter of length 5 ms. We located the first local maximum following the stimulus onset that was larger than the average spontaneous activity (100 ms before the onset of the stimulus) plus seven times the standard deviation of the spontaneous activity as well as having a large enough prominence (larger than 5% of the standard deviation of the spontaneous activity; see https://www.mathworks.com/help/signal/ug/prominence.html). We then located the local minimum preceding the local maximum, also requiring its prominence to be larger than 5% of the standard deviation in the spontaneous activity. Finally, we fitted a quadratic polynomial to the firing rates between these two and interpolated the timepoint in which this polynomial reached 25% of the local maximum. Finally, to correct for the “smearing” of the activity in time as a result of the smoothing filter, the estimated onset was shifted by 1.5 ms (30% of the 5 ms window used for smoothing). This timepoint was considered as the onset latency of the unit.

#### Best frequencies

To estimate the BF of the auditory-responsive units from the FS stimulation protocol, we used a modification of the approach of Guo et al. (2012). The frequency that elicited the largest response in a 100 ms window poststimulus, for the three lowest attenuations, was used as the BF.

#### Unit selection for the analysis of SSA

Units selected for the stimulus-specific adaptation (SSA) analysis had to show significant responses using the inhomogenous Poisson significance test (*p* < 0.01) as well as a minimum response of at least five spikes in total to at least one of the frequencies used in the rare condition. Responses to each frequency in each condition were quantified by the average spike count in a time window of 40 ms poststimulus. Overall, 666 units from the insulo-parietal field and 355 from A1 were analyzed here.

#### Adaptation in narrow frequency channels (ANFC)

Units were used in this analysis if they were selected for the SSA analysis and in addition the average response in the deviant condition was larger than the average response in the standard condition. Overall, 444 insulo-parietal and 282 A1 units were used here. The model is based on Taaseh et al. (2011). This model predicts the response to frequency $f_{0}$, by estimating the resources used in a narrowly tuned adaptation channel around $f_{0}$. The model has two parameters: *σ* dictates the width of the adaptation channels, and *τ* is the time constant of the exponential resource recovery between stimulus presentations. It can be shown (Taaseh et al., 2011) that given a fixed ISI d of a set of frequencies {f} played randomly with probabilities $\left\{p_{f}\right\}$, the expected response to frequency $f_{0}$ is as follows:

$$E(R_{\;f_{0}})=A\frac{1}{1-(1-\sum_{f}p_{f}U(\;f_{0},f))\delta },$$

where *A* is the ideal unadapted response to $f_{0}$, $U(\;f_{0},f)\;$ is the kernel that defines the adaptation channel centered on frequency $f_{0}$, and $\delta =e^{-\frac{d}{\tau }}$. In our analysis, we used $U(\;f_{0},f)=e^{-\frac{\log\;{\left(\frac{\;f}{\;f_{0}}\right)}^{2}}{2\sigma^{2}}}$. We normalized the responses to the rare responses and therefore assumed that *A* = 1. We then optimized the parameters *σ* and *δ* using MATLAB's lsqnonlin function to fit the responses in the other SSA conditions. The fit was computed for each unit separately.

## Results

Extracellular spiking activity was recorded in 15 rats from the left posterior insular cortex (Ins) and the nonprimary somatosensory areas adjacent to Ins dorsally. The full extent of this auditory-responsive region is referred to here as the insulo-parietal field. In addition, auditory units were also recorded in the primary auditory cortex (A1), secondary auditory fields (AC), MGB, and IC, in a separate group of five rats for each region, using the same set of sounds. In the rat brain atlas, A1 corresponds to Au1, and AC includes both AuD and AuV (Paxinos and Watson, 2007). The somatosensory areas are marked as S2 (Paxinos and Watson, 2007), although they contain at least two separate fields, the PV field which borders the posterior insula and S2 proper (Fabri and Burton, 1991; Remple et al., 2003).

### Location of auditory units in the insulo-parietal auditory field in the rat

Recordings of neuronal activity were obtained using two types of electrodes: monopolar tungsten electrodes (*n* = 7) and Neuropixels silicon probes (*n* = 8, 23 independent brain penetrations; Fig. 1). Neuropixels probes facilitated high-yield recordings of neuronal activity from multiple units in Ins and S2 with each penetration.

To determine the location of auditory units, we used the consecutive coronal brain sections covering the area with visible electrode tracks. Based on the histological material, we identified which neurons were recorded in the Ins and which in the parietal somatosensory fields, their cortical layers, and the subregions of Ins: granular insular cortex (GI), dysgranular insular cortex (DI), and agranular insular cortex (AI; Fig. 1*A*). The numbers of auditory and nonauditory units recorded in all rats across these structures are listed in Table 2. We drew the borders between Ins subregions based on the presence and morphology of granular layers and their relative position with respect to other brain structures (i.e., claustrum, endopiriform nucleus, piriform cortex, external capsule, anterior commissure, septum, ventricles, thalamic nuclei, and other structures depending on anteroposterior coordinates of given brain section). All histological reconstructions and divisions were made in accordance with the rat brain atlas (Paxinos and Watson, 1997, 2007).

**Table 2.** The numbers of all detected units and auditory cells in the secondary somatosensory cortex (S2) and insular cortex (Ins) were obtained using both Neuropixels and tungsten electrodes

	S2	Ins	Ins			Ins/Par field
			GI	DI	AI	
All units						
Neuropixels and tungsten electrodes	2,099	2,119	1,338	724	57	4,218
Neuropixels electrodes	1,910	1,884	1,170	670	44	3,794
Tungsten electrodes	189	235	168	54	13	424
Neuropixels/tungsten unit numbers ratio	10	8	7	12	3	9
Auditory units						
Neuropixels and tungsten electrodes	799	939	699	230	10	1,738
Percentage of auditory units by brain region	38%	44%	52%	32%	18%	41%
Neuropixels electrodes	670	781	572	202	7	1,451
Percentage - Neuropixels electrodes	35%	41%	49%	30%	16%	38%
Tungsten electrodes	129	158	127	28	3	287
Percentage: tungsten electrodes	68%	67%	76%	52%	23%	68%
Neuropixels/tungsten unit numbers ratio	5	5	5	7	2	5

Counts are divided by Ins subregions: GI, DI, and AI, as well as for the whole parieto-insular field. Additionally, the percentages of auditory units for Neuropixels and tungsten electrodes are reported. The lower percentage of auditory units in the neuropixels recordings are likely due to the much higher yield of these recordings, which include often units with very low firing rates, too low to demonstrate any statistical relationships with the sound presentations.

We recorded a total of 4,218 units in the insulo-parietal field, out of which 1,738 (41%) were classified as auditory neurons (see Materials and Methods for criteria). The median anteroposterior coordinate of the electrode tracks was −0.48 mm posterior to the bregma (max, +0.24 mm; min, −1.56 mm; *n* = 48 electrode tracks). Auditory neurons were concentrated in ventral S2 and in the adjoining GI, where up to half of the neurons showed some auditory responses (Fig. 1*B*). The abundance of auditory responses decreased gradually outside this band, both dorsally in the nonprimary somatosensory fields and ventrally in the DI and AI regions of Ins. We recorded 799/2,099 (38%) auditory units in the nonprimary somatosensory fields. In Ins there were 939/2,119 (44%) auditory units, of which GI had 699/1,338 (52%); DI had 230/724 (32%); and AI had 10/57 (18%).

We compared these results with neural responses recorded previously in three main stations of ascending auditory system: the IC (midbrain), the MGB (diencephalon), and the primary and secondary auditory cortex (A1 and AC, telencephalon), each in five rats (Harpaz et al., 2021). The units were analyzed in the same way as those in the insulo-parietal field. We recorded 1,759/2,275 (77%) auditory units in IC; 1,684/2,149 (78%) auditory units in MGB; 1,304/2,502 (52%) auditory units in A1, and 1,401/3,117 (45%) in AC (Table 3). Thus, the fraction of auditory units at the center of the insulo-parietal field was comparable to that found in the core ascending auditory pathway.

**Table 3.** The numbers of all detected units and auditory cells in the structures of the ascending auditory system: the IC (all subdivisions), the MGB (all subdivisions), the primary auditory cortex (A1), and surrounding fields (AC, non-A1 subdivisions: Aud/Auv)

Ascending auditory system	IC	MGB	A1	AC
	All subdivisions	All subdivisions		Non-A1 subdivisions
All units	2,275	2,149	2,502	3,117
Auditory units	1,759	1,684	1,304	1,401
The percentage of auditory units by the brain region	77%	78%	52%	45%

The percentage of auditory cells per tested region is provided.

### Onset latencies

The majority of auditory units recorded in the insulo-parietal field had short-onset latency, in response to both BBN (median, 16.8 ms; IQR, 12.3–27.3 ms) and to pure tones (median, 18.4 ms; IQR, 14.0–25.1 ms; Figs. 1*C*,*D*). Onset latency was surprisingly short: 34% (393/1,153) of onset latencies computed for neurons in the insulo-parietal field were equal or shorter than the first percentile onset values in A1, computed using the same procedures. We observed similar distribution of onset latencies for units recorded in the insulo-parietal field with tungsten and Neuropixels electrodes across all rats (Fig. 1*E*).

The onset latencies differed among the different anatomical subdivisions (*F* = 84.3; df = 2; *p* = 6.4 × 10^−35^; ANOVA test; units in the somatosensory fields lumped as “S2” below, in GI and in DI). Shorter latencies were observed in S2 and GI, while longer latencies were observed more ventrally in DI. The median latency of S2 units was 15.2 ms (IQR 12.5–19.8 ms). In GI, the median latency was 19.4 ms (IQR 13.8–31.2 ms), and in DI, the median latency was 34.1 ms (IQR 20.4–56.5 ms). All differences were significant: both Ins regions had significantly longer median onset latencies than S2 units (GI vs S2, rank sum = 315,898; *z* = 7.9285; *p* = 2.2 × 10^−15^ and DI vs S2, rank sum = 46,629; *z* = 10.6; *p* = 3.9 × 10^−26^, two-sided Wilcoxon rank-sum test), while units in the DI region had significantly longer latencies than units in the GI region of Ins (rank sum = 39,483; *z* = 6.91; *p* = 4.9 × 10^−12^; two-sided Wilcoxon rank-sum test). Minimum latencies of S2 and GI units were similar, but GI had in addition a population of longer-latency neurons. DI lacked neurons with relatively short-onset latencies (<20 ms) compared with GI and S2 ( $\chi^{2}=70.9$; df = 1; *p* = 3.7 × 10^−17^).

**Figure 2.**
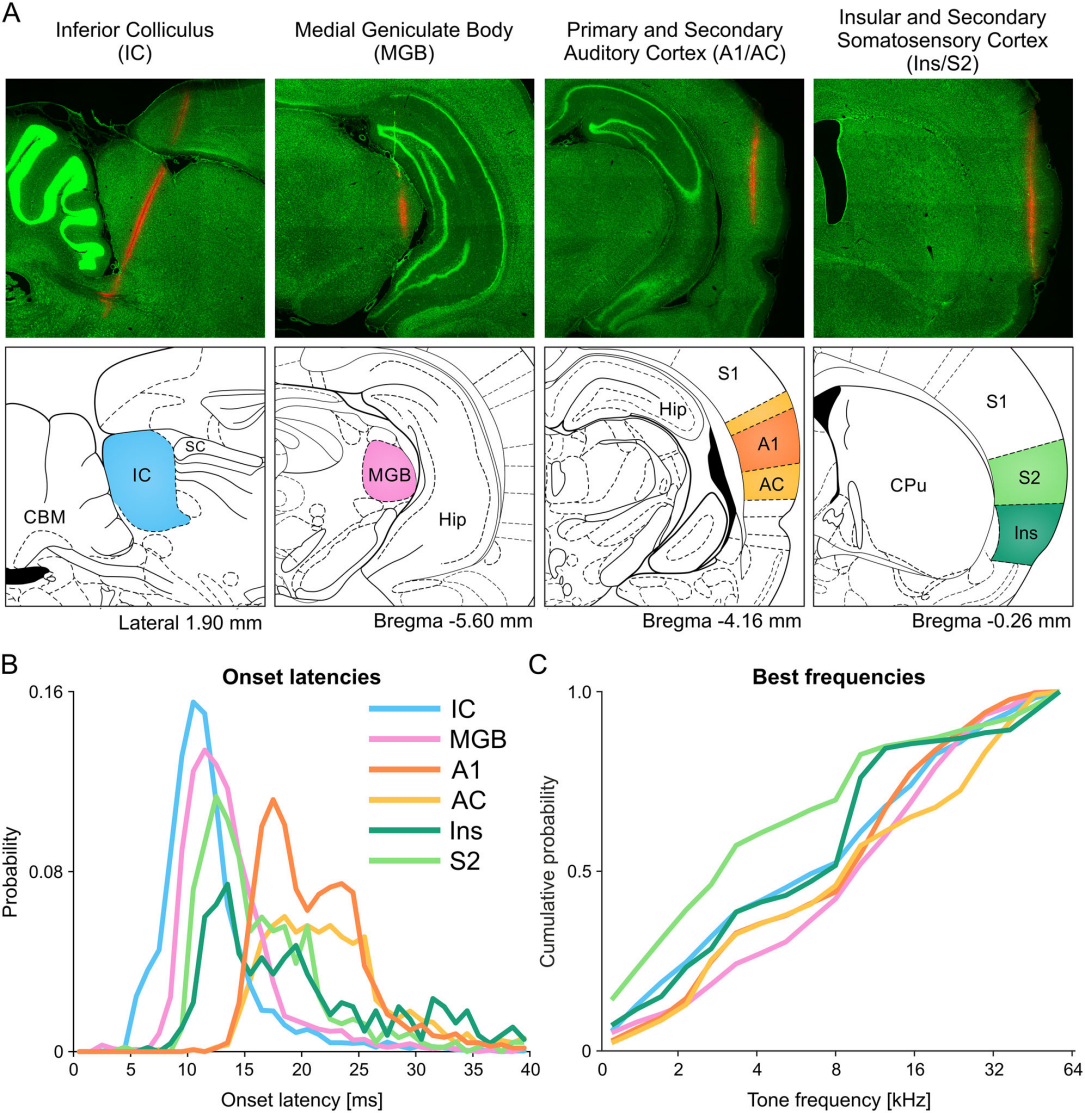
Onset latencies and BFs across structures of ascending auditory system and the insulo-parietal field. ***A***, Representative fragments of sagittal and coronal brain sections with Neuropixels probes tracks in multiple structures of the auditory system [from left to right: the IC, MGB, primary and secondary auditory cortices (A1/AC), and the insulo-parietal field]. The lower row consists of schematic drawings depicting the borders of the respective structures. ***B***, Probability distribution of onset latencies for all units in these auditory structures. Note the short-latency units in the insulo-parietal field, whose responses precede that of the units in A1. The onset latencies displayed here include only the short-latency peak in each structure, resulting in differences in the total area of the different histograms—the cortical populations included also long-latency neurons which are not displayed here, resulting in a smaller area of the short-latency peak. ***C***, Cumulative probability of BFs in the same auditory structures. Note the higher probabilities for units with BFs 16–32 kHz in the insulo-parietal field compared with the core auditory stations. A1, primary auditory cortex; AC, secondary auditory cortices; CBM, cerebellum; CPu, caudate–putamen; Hip, hippocampus; IC, inferior colliculus; Ins, insular cortex; MGB, medial geniculate body; S1, primary somatosensory cortex; S2, nonprimary somatosensory fields as defined in Paxinos and Watson (1997); SC, superior colliculus.

**Figure 3.**
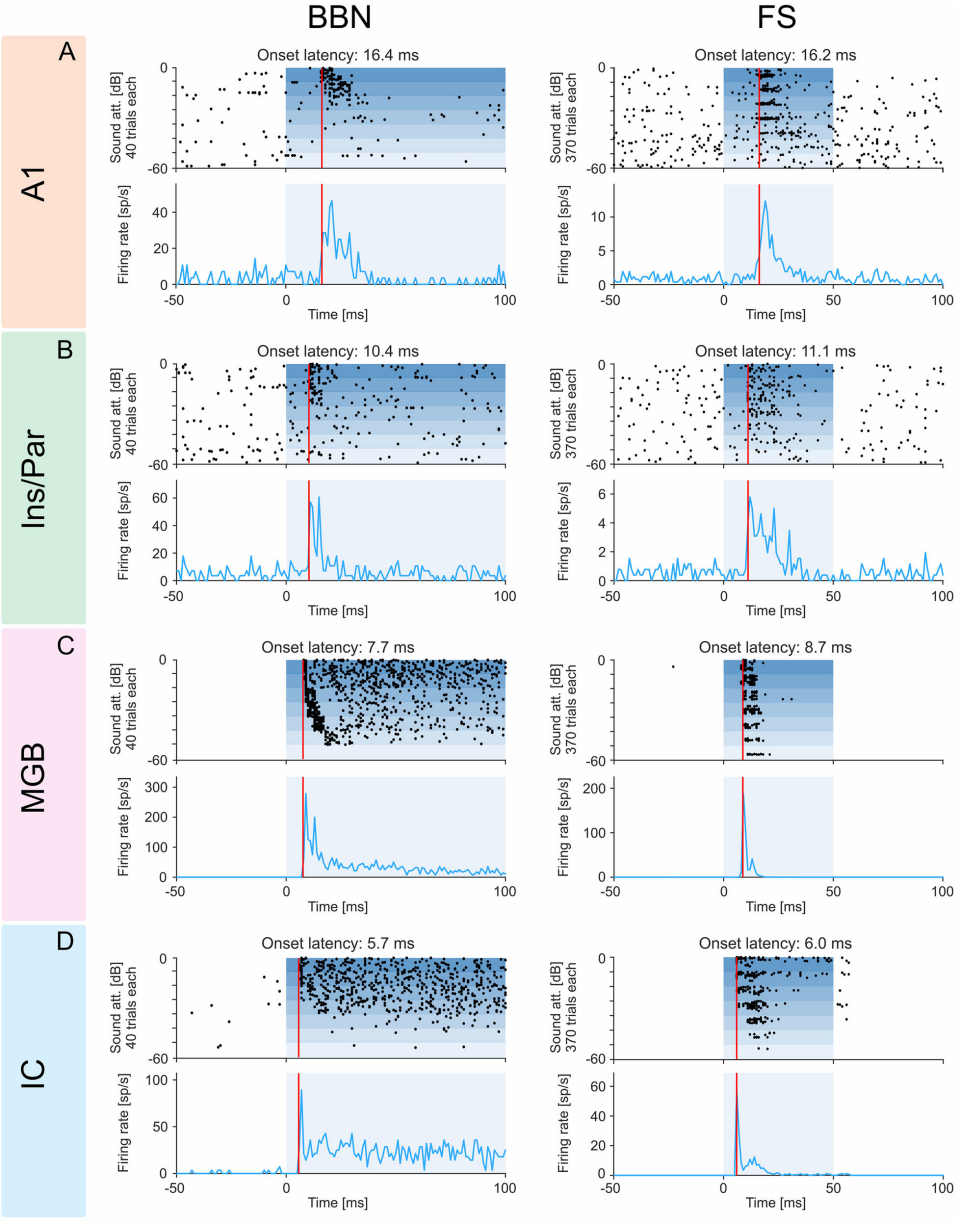
Exemplary responses of units to BBN and frequency sweeps (FS). Representative neuronal responses to BBN (280 trials, 10 for each sound level, left column) and FS (370 trials, 10 sound levels for 37 frequencies, right column) of units recorded in (***A***) the primary auditory cortex (A1), (***B***) the insulo-parietal field (Ins/Par), (***C***) the MGB, and (***D***) the IC. The histograms below each raster show firing rates averaged over all trials. The red vertical line represents onset latency as objectively determined (see Materials and Methods for details). Shading represents stimulus duration (BBN bursts were 200 ms long; only the initial 100 ms are shown, for consistency with the FS responses). The examples illustrate the general trend of average latencies getting longer in the following order: IC→MGB→insulo-parietal field→A1.

We compared these latencies with those measured in IC, MGB, and AC using the same methods and found that onsets were significantly different among the different brain regions (*F* = 803; df = 5; *p* = 0; ANOVA test; Fig. 2*A*,*B*). As expected, IC units typically had the shortest-onset latencies in our samples (median, 11.2 ms; IQR, 9.6–13.1 ms), followed by MGB units (median, 12.8 ms; IQR, 11.0–15.5 ms). Surprisingly, auditory neurons in the insulo-parietal field had significantly shorter-onset latencies (median, 17.6 ms; IQR, 13.4–26.1 ms) than in A1 (median, 20.3 ms; IQR, 17.6–24.0 ms; rank sum = 1,229,470; *z* = −9.46; *p* = 3.1 × 10^−21^; two-sided Wilcoxon rank-sum test) and surrounding fields (median, 24.1 ms; IQR, 19.4–42.1 ms; rank sum = 987,401; *z* = −17.7; *p* = 9.3 × 10^−70^; two-sided Wilcoxon rank-sum test). Responses of representative neurons recorded in each of these regions are presented in Figure 3.

### Frequency tuning

Frequency tuning was evaluated based on the responses to pure tones (1–64 kHz, 6 tones/octave; Figs. 1*F*,*G*, 2*C*). Best frequencies were significantly different among the various brain regions (*F* = 29.6; df = 5; *p* = 6.3 × 10^−30^; ANOVA test). Auditory-responsive units recorded in the insulo-parietal field were preferably tuned to tones of frequencies below 16 kHz when compared with units recorded in either A1 or surrounding auditory fields ( $\chi^{2}=136;\;\;\mathrm{df}=1$; *p* = 2.0 × 10^−31^), as depicted in representative example (Fig. 1*F*) and for all units (Fig. 1*G*). Median best frequency in the insulo-parietal field was 4.8 kHz, and IQR was 2.0–10.1 kHz (in A1 and surrounding fields, median 9.0 kHz, IQR 2.8–20.2 kHz).

### SSA

SSA is the decrease in the responses to a commonly presented stimulus (“the standard”) which is not, or only partially, generalized to another, rare frequency (“the deviant”). Neurons throughout the auditory system show SSA. We followed here a methodology that characterizes context dependence of the responses to pure tones in a fair generality (Taaseh et al., 2011). Two tone frequencies were selected within the frequency range of the units recorded in each site, and each of these frequencies was tested in six different conditions (see Materials and Methods for details). Three of these conditions consisted of sequences composed of the two frequencies only: standard (95% of tone presentations in the sequence), deviant (5% tone presentations), and equal (50% tone presentations). The “rare” condition (with intertone intervals of many seconds) provided estimates of the least adapted responses. The other two conditions were multitone sequences in which each of the two tested frequencies were presented 5% of the time. In one of these sequences, the other tones covered a narrow frequency range (“diverse narrow”) and in the other sequence they covered a wide frequency range (“diverse broad”).

There are a number of relevant contrasts measured using these sequences. The responses to tones when deviants are often larger than when they are standard; this contrast is termed SSA. The rare condition provides an estimate of the unadapted responses, while the diverse conditions, in particular the diverse broad, provides an estimate of the responses in a sound context with minimal predictability: while in oddball sequences the deviant sounds violate the expectation for the standard sound to appear next, in the multitone sequences, no expectations can be formed for the next tone frequency. The two multitone sequences differ however in the amount of across-frequency adaptation that may occur (Taaseh et al., 2011). Responses to deviants that are larger than to the same tone frequency in the diverse broad condition are sometimes used as evidence for “deviance senstivity,” in that the violation of the prediction (by the deviant tone) produces a response that is larger than when no prediction violation occurred (in the diverse broad sequence).

SSA and deviance detection have been extensively studied in the ascending auditory system (Taaseh et al., 2011; Hershenhoren et al., 2014; Parras et al., 2017). Thus, measuring these response properties in the insulo-parietal auditory field is important for understanding its relationships to other auditory stations, both cortical and subcortical.

**Figure 4.**
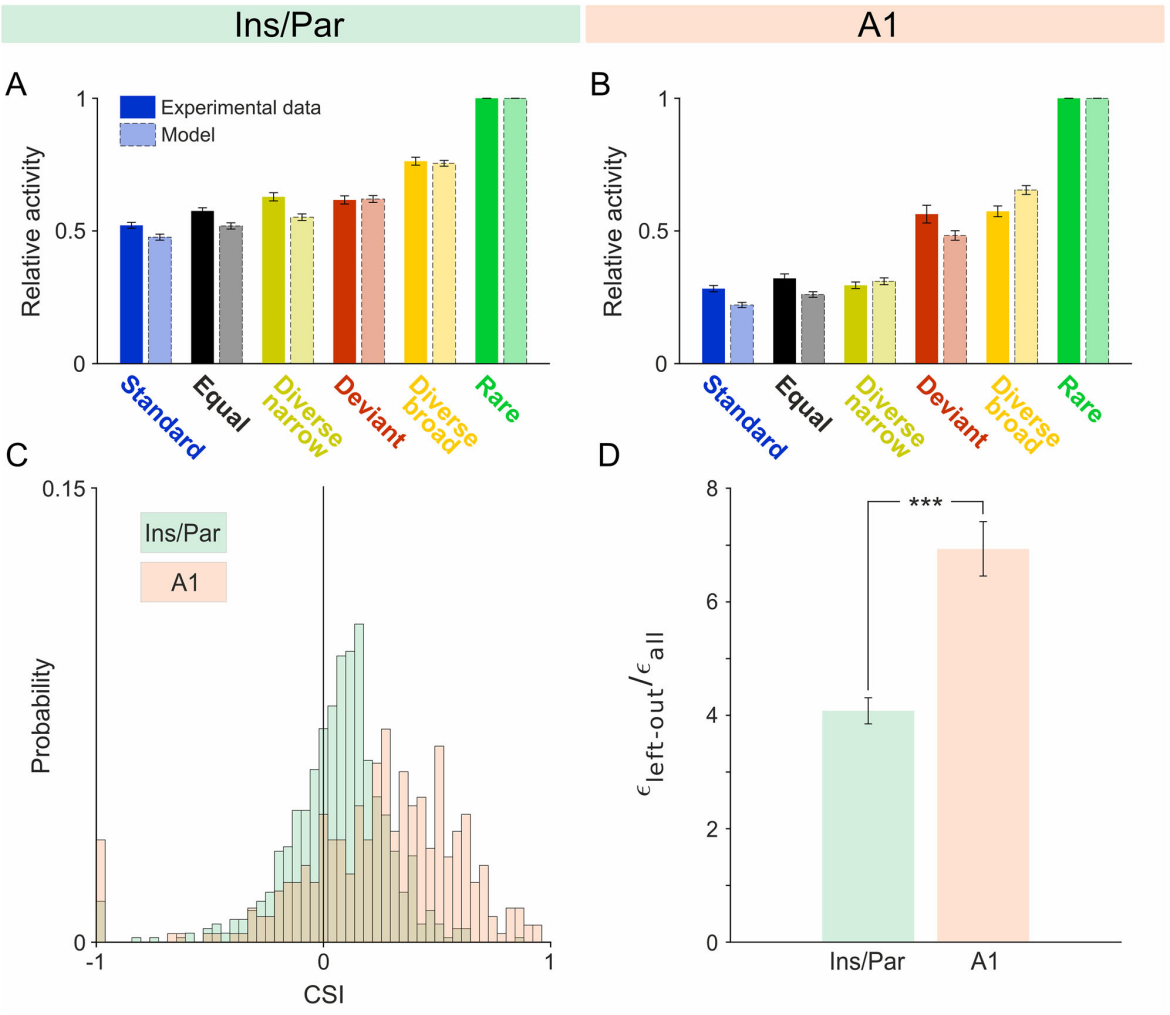
SSA: Insulo-parietal field versus A1. Panels ***A*** and ***B*** show the mean responses ± SEM of MUA (solid-color bars) and the model-predicted responses (faded-color bars outlined by dashed lines) for the insulo-parietal field and A1, respectively. As previously reported, in A1, responses to deviant tones in diverse broad and oddball sequences were indicative of deviance sensitivity due to larger-than-expected deviant responses. In contrast, in the insulo-parietal field, diverse broad responses exceeded deviant responses, demonstrating better alignment with the model. In ***C***, overlaid histograms of CSI for the insulo-parietal field and A1 are presented. Overall, we observed a higher proportion of positive CSI values for units in A1 than in the insulo-parietal field. ***D***, Deviant prediction error for Ins versus A1. Plotted here is the mean ± SEM of the normalized error values obtained from the leave-one-out analysis of the NFAC model. Error values are calculated as the squared difference of the predicted deviant response from the model fitted on all conditions excluding the deviant response and the measured response, both normalized to the responses at the rare condition. The normalization is by the average squared error of the model when fitted on the complete set of responses. The normalized errors in the insulo-parietal field were significantly smaller than in A1.

To quantify how presentation probability affects tone response (Fig. 4*A*,*B*), we calculated the common SSA index (CSI) between the deviant and standard responses to characterize the average effect of adaptation for this specific pair of frequencies as follows:

$$\mathrm{CSI}=\frac{d(\;f1)+d(\;f2)-s(\;f1)-s(\;f2)}{d(\;f1)+d(\;f2)+s(\;f1)+s(\;f2)},$$

where *d*(*f*1) and *s*(*f*1) represent the average responses to frequency *f*1 when it was deviant and standard, respectively. The average CSI in the insulo-parietal field (mean, 0.054; ±0.009 SE) was significantly smaller than in A1 (mean, 0.242; ±0.019 SE; *t* = −9.83; df = 1,019; *p* = 7.3 × 10^−22^; two-sample *t* test; Fig. 4*C*).

We also calculated the frequency-specific SSA index (SI) as follows:

$$\mathrm{SI}1=\frac{d(\;f1)-s(\;f1)}{d(\;f1)+s(\;f1)}\;\;\mathrm{and}\;\;\mathrm{SI}2=\frac{d(\;f2)-s(\;f2)}{d(\;f2)+s(\;f2)}.$$

Overall, the neurons in the insulo-parietal field had a smaller number of cases where both SI1 and SI2 were positive (317/666, 48%) than in A1, where this effect was stronger (194/355, 55%; $\chi^{2}=4.99;\mathrm{df}=1;\;p=0.025$). Thus, insulo-parietal units showed a weaker effect of stimulus probability on their responses than A1 units.

To quantify deviance sensitivity and explain the adaptation profiles in the insulo-parietal field and in A1, we used a previously established model based on ANFC (Taaseh et al., 2011). The model assumes that the neural responses to any frequency depend on the available resources in a narrowly tuned adaptation channel centered on it. These resources are depleted by tone presentations according to how far they are from the center of the adaptation channel and recover exponentially between stimulus presentations. The model has two parameters, one for the width of the adaptation channels and the other for the recovery time constant of the resources between tone presentations (see Materials and Methods). The width of the adaptation channels determines the effects of presentations of a tone of one frequency on the responses to tones of another frequency through the “adaptation load”—the average amount of resources depleted within the adaptation channel centered on the TF. The adaptation load differed for the different sequences used here (Taaseh et al., 2011; see Materials and Methods for detail). The model was fitted to units in the insulo-parietal field and in A1, and predictions fitted well to the measured responses (Fig. 4*A*,*B*).

We used the model to compare the deviance sensitivity of neurons in the insulo-parietal field relative to that of neurons in A1 using the method of Taaseh et al. (2011). Deviance sensitivity is tested by comparing the responses to the same frequency in the diverse broad and in the deviant conditions. In both conditions, the test frequencies are played with the same probability (5%). However, in the diverse broad condition, all frequencies were presented with low probabilities, so that none was special (no “deviance”). This is in contrast with the deviant condition, in which the standard may generate expectations that are violated by the presentations of the deviant tone (Jacobsen et al., 2003).

The ANFC model predicts responses that are smaller to deviants than to the same sounds in the diverse broad sequence, because for the deviant, all other tone presentations consist of the standard tone which is about half an octave away, while in the diverse broad sequence many of the other tone presentations are farther away from the tone of interest [see Taaseh et al. (2011) for a quantitative treatment]. In A1, contrary to the predictions of the model, diverse broad responses and deviant responses were similar (*t* = −0.33; df = 354; *p* = 0.74), reproducing previous results (Taaseh et al., 2011; Hershenhoren et al., 2014; Polterovich et al., 2018; Fig. 4*B*, red and yellow bars; note discrepancies with the model predictions, in light colors). We have previously interpreted the similarity in responses to deviant and diverse broad in A1 as indicating the presence of deviance sensitivity, since the responses to the deviant condition were larger than expected by the ANFC model (red bar larger than the light red bar), suggesting the presence of an additional response component that was sensitive to deviance.

On the other hand, in the insulo-parietal field, diverse broad responses were significantly larger than deviant responses (*t* = −9.03; df = 665; *p* = 1.7 × 10^−18^), better fitting the ANFC model (Fig. 4*A*, red and yellow bars). Furthermore, in the insulo-parietal field, the normalized differences between the responses to diverse broad and deviant were significantly larger than the differences observed in A1 (*t* = 2.8; df = 1,014; *p* = 0.0057). Thus, deviance sensitivity in Ins is weaker than in A1.

To further test deviance sensitivity in our data, we ran a leave-one-out analysis, in which we fitted the model to the responses to all tone sequences except for the deviant condition and compared the predicted responses in the deviant condition to the measured ones. There were significantly larger errors in predicting the deviant response of units in A1 than for units in the insulo-parietal field (Fig. 4*D*; rank sum = 142,545; *z* = −6.84; *p* = 7.7 × 10^−12^; two-sided Wilcoxon rank-sum test), supporting the conclusion that units in the insulo-parietal field showed less deviance sensitivity than units in A1.

### The insulo-parietal auditory field

We describe here auditory-driven neurons in the insular cortex and the adjacent secondary somatosenaory cortex. Remarkably, these auditory-driven neurons were recorded, under anesthesia, outside of the core auditory fields in rats, showing that these responses are purely sensory. Auditory neurons were present in the posterior insular cortex, particularly in the GI, extending medially to the adjacent nonprimary somatosensory fields.

Units throughout the insulo-parietal field shared response properties: (1) We describe a mediolateral gradient of response latencies. In the medial part of the field, units had on average shorter-onset latencies to BBN and pure tones than more laterally, with the longest latencies in that part of the auditory field that lies in DI. Remarkably, some neurons had shorter-onset latencies than the shortest latencies recorded using the same methods in the primary and secondary auditory cortices. (2) The insulo-parietal auditory units were preferentially tuned to relatively low frequencies (<16 kHz). (3) The habituation profiles of neurons in the insulo-parietal field were subtly different from those found in the primary auditory cortex, with greater concordance with a simple adaptation model and in consequence less deviance sensitivity.

These observations establish the presence of a fast auditory information stream through the insulo-parietal field, in parallel to the usual primary pathway to the primary auditory cortex and its surrounding fields.

## Discussion

### The insulo-parietal auditory field in rodents

Many studies of the parcellation of auditory fields in rats ignored the more anterior areas studied here (Polley et al., 2007; Nieto-Diego and Malmierca, 2016). Nevertheless, previous research has identified sound-responsive neurons in the insular and somatosensory cortices. In rats, Rodgers et al. (2008) used epipial recordings to identify the cortical area that responds to sound in the posterior insula, showing that it is anatomically separated from the auditory cortices in the temporal lobe by the insular somatosensory field (Brett-Green et al., 2004; Rodgers et al., 2008). Kimura et al. (2010) used precise but low-yield recording and tracing methods, followed by histological analysis, to establish the presence of auditory responses in the posterior insular cortex. The anterior–posterior location and extent of this field are largely consistent in these two studies and in ours.

We establish the presence of auditory neurons not only in the insular cortex but also, more medially, in the ventral portion of the adjacent nonprimary somatosensory fields [likely PV; lumped with S2 in Paxinos and Watson (2007) ]. Multimodal somatosensory–auditory units have been reported on the border between the parietal and temporal lobes (Brett-Green et al., 2003, 2004; Wallace et al., 2004), and some studies have described neuronal responses to sounds in somatosensory cortices of rats (Brett-Green et al., 2003; Maruyama and Komai, 2018) and of mice (Carvell and Simons, 1986; Kwon et al., 2016; Zhang et al., 2020; Godenzini et al., 2021). However, the main two studies of the auditory insular cortex in rats, Rodgers et al. (2008) and Kimura et al. (2010), attributed auditory function exclusively to the insular cortex. The use of Neuropixels probes allowed us to record significantly more units simultaneously across both areas, establishing the presence of auditory neurons in the ventral part of adjacent nonprimary somatosensory fields, in addition to the previously reported auditory neurons in the insular cortex.

Among rodents with lissencephalic brains, the separation of the insulo-parietal field from the more posterior core auditory fields is well documented only in rats (Brett-Green et al., 2004; Rodgers et al., 2008; Krubitzer et al., 2011). In mice, an insular auditory field exists, but is continuous with the anterior auditory field (AAF) in the ventrorostral direction (Sawatari et al., 2011; Guo et al., 2012; Gogolla et al., 2014; Takemoto et al., 2014; Tsukano et al., 2017). The anatomical separation in rats between the insular auditory field and other auditory fields is a distinct advantage relative to mice, where it is harder to distinguish between these fields. Rats therefore offer a valuable model for investigating the insular auditory field.

Evidence for thalamic inputs from the ventral, primary, division of the MGB to the insular cortex has been reported in cats (Rose and Woolsey, 1958). In mice, the ventral division of the MGB provides direct input to the insular cortex through a population distinct from that projecting to the primary auditory cortex as well as to the AAF (Takemoto et al., 2014).

In rats, the posterior insular cortex is innervated by nonlemniscal auditory thalamic nuclei, including the medial division of the MGB and the suprageniculate thalamic nucleus, as well as the nonspecific posterior intralaminar thalamic nuclei (Guldin and Markowitsch, 1983; LeDoux et al., 1985; Linke, 2000). Evidence for direct inputs from the ventral division of the MGB to the insular cortex in rats is lacking. In addition to direct auditory thalamic inputs, the posterior insular cortex receives inputs from the ventral auditory field which is largely innervated by the dorsal division of the MGB (Kimura et al., 2007).

The nonprimary somatosensory fields [PV and S2, marked together as S2 in Paxinos and Watson (2007)], where we found auditory neurons in rats, are less explored in terms of auditory inputs. The ventral part of the nonprimary somatosensory cortex, PV, has been suggested to contain a complete representation of the contralateral body surface. This has been first demonstrated using microelectrode unit recordings in gray squirrel (Krubitzer et al., 1986). Subsequent studies in rats followed the division established by Krubitzer et al. (1986), similarly identifying an S2 and a PV subregion (Fabri and Burton, 1991; Remple et al., 2003; Benison et al., 2007).

In a number of species, PV (and potentially also S2) receive auditory inputs. In cats, an area of secondary somatosensory cortex analogous to PV receives projections from the medial division and the suprageniculate nuclei of the MGB (Niimi et al., 1987). A partial overlap between PV and the insular auditory field, which is directly innervated by the ventral division of the MGB, has been described in mice (Nishimura et al., 2015). Thus, while evidence for innervation of PV by the ventral division of the MGB is lacking in rats, it appears to exist in mice.

In our study, we recorded neural activity in the most ventral part of the nonprimary somatosensory cortex adjacent to the GI. This region is likely to correspond to the head representation within the PV region of Remple et al. (2003). Further functional studies are needed to delineate the somatosensory representations in the auditory-responsive zone of these nonprimary somatosensory fields.

### Sound responses in the insulo-parietal auditory field

We show two major differences between the auditory responses in the core fields and in the insulo-parietal field. The first difference is the relatively simple adaptation profile of auditory neurons located in the insulo-parietal field (Fig. 4). Responses were substantially reduced in all sequences relative to the least adapted condition (“rare”), and differences between the different conditions were smaller than in A1. Indeed, a simple adaptation model predicted better the responses to deviant tones in the insulo-parietal field than in A1. We have previously interpreted the discrepancy between the model and deviant responses in A1 as an indication for true deviance sensitivity (Taaseh et al., 2011; Hershenhoren et al., 2014; Polterovich et al., 2018). In the insulo-parietal field, in contrast, the responses to tone sequences seem to be largely governed by adaptation, without true deviance sensitivity. Nieto-Diego and Malmierca (2016) suggested that SSA to tones is stronger in nonprimary auditory fields than in primary fields (Duque et al., 2015). Our results here [as well as in Polterovich et al. (2018)] suggest a more nuanced view, in which different secondary fields may show different profiles of SSA and deviance sensitivity. Further studies are required to resolve this issue.

The most surprising result of this paper is the observation of units in the insulo-parietal field whose onset latencies to BBN and pure tones, while longer than in subcortical stations, were shorter than the shortest response latencies recorded in the primary and secondary auditory cortices using exactly the same methods (Figs. 2, 3).

Consistent with our data, Rodgers et al. (2008) reported that the latencies of the P1 and N1 peaks in the insular auditory field were significantly shorter than the latencies for the same components in the AAF, suggesting that the insular auditory field does not rely on intracortical projections from the AAF to access auditory inputs. Kimura et al. (2010) reported the lowest onset latency of ∼10 ms (on average 12.7–19.8 ms) in rats, comparable to the shortest-onset latencies in A1. Our data are the first demonstration of response latencies in the insulo-parietal field that are shorter than those found in A1.

In mice, as well, responses in the insular cortex are remarkably fast. For example, Guo et al. (2012) observed latencies of ∼10 ms on average in the insular auditory field of mice. Similarly, Sawatari et al. (2011) observed the fastest cortical voltage-sensitive dye responses to 4 kHz tones in the insular auditory field.

The medial division of the MGB has the largest neurons across the auditory thalamus (Winer and Morest, 1983). These neurons receive inputs from the lateral and rostral cortex of the IC (Malmierca et al., 2015) but are also directly innervated by axons from the dorsal cochlear nucleus (Anderson et al., 2006). It is possible that the pathway from DCN to the medial MGB, bypassing the IC, may be the source for the short-latency responses in the insulo-parietal field (Kline et al., 2022).

The short latencies of the insulo-parietal auditory neurons in rats indicate the presence of a fast stream of auditory information to the cortex, parallel to the leminscal pathway through A1. Our results suggest the presence of multiple, parallel information pathways from the thalamus to the cortex. It is tempting to hypothesize that this pathway is specialized for processing communication sounds, particularly in view of the dominant low-frequency representation in our results. Indeed, vocalizations of species that prey on small rodents typically involve energy below 4 kHz (Brown et al., 1978; Farley et al., 1992). Furthermore, rats communicate in the audible range (<16 kHz), and these low-frequency calls are often associated with pain-related behavior or produced in response to noxious stimuli (Han et al., 2005). These findings are particularly intriguing in view of the recent description of a parallel thalamocortical circuit for the processing of speech in humans (Hamilton et al., 2021; Kurteff et al., 2024).

## Data Availability

The associated dataset can be found at https://data.mendeley.com/preview/nkk7rjfvhw?a=7a98f29e-5615-4894-9a13-6361ea4d34c1.
